# The High-Pressure Superconducting Phase of Arsenic

**DOI:** 10.1038/s41598-018-20088-8

**Published:** 2018-02-14

**Authors:** Prutthipong Tsuppayakorn-aek, Wei Luo, Rajeev Ahuja, Thiti Bovornratanaraks

**Affiliations:** 1 Extreme Conditions Physics Research Laboratory (ECPRL) and Physics of Energy Materials Research Unit (PEMRU), Department of Physics, Faculty of Science, Chulalongkorn University, Bangkok, 10330 Thailand; 2 Thailand Center of Excellence in Physics, Commission on Higher Education, 328 Si Ayutthaya Road, Bangkok, 10400 Thailand; 3 Condensed Matter Theory Group, Department of Physics, Uppsala University, Box 530, S-751 21 Uppsala, Sweden; 4 Department of Materials and Engineering, Applied Materials Physics, Royal Institute of Technology (KTH), SE-100 44 Stockholm, Sweden

## Abstract

*Ab initio* random structure searching (AIRSS) technique is predicted a stable structure of arsenic (As). We find that the body-centered tetragonal (bct) structure with spacegroup *I*4_1_/*acd* to be the stable structure at high pressure. Our calculation suggests transition sequence from the simple cubic (sc) structure transforms into the host-guest (HG) structure at 41 GPa and then into the bct structure at 81 GPa. The bct structure has been calculated using *ab initio* lattice dynamics with finite displacement method confirm the stability at high pressure. The spectral function α^2^*F* of the bct structure is higher than those of the body-centered cubic (bcc) structure. It is worth noting that both bct and bcc structures share the remarkable similarity of structural and property. Here we have reported the prediction of temperature superconductivity of the bct structure, with a *T*_*c*_ of 4.2 K at 150 GPa.

## Introduction

The group-V element is one of central interest in a discipline in high-pressure physics and continues to attract a lot of attention as it has an incommensurate host-guest (HG) structure. The existence of a HG structure in the group-V elements is observed in As-III, Sb-IV, and Bi-III^1^. They are a common high-pressure structure among the elemental metals. With increasing pressure, for As, a HG structure transforms into a body-centered cubic (bcc) structure at pressure 97 GPa. For Sb, a HG structure transforms into a bcc structure at pressure 28 GPa. For Bi, a HG structure transforms into a bcc structure at pressure 8 GPa. It is worth noting that the bcc crystal structure of them is the most stable structure at the highest pressure.

Arsenic (As) maintains the bcc crystal structure up to at least 122 GPa^2^, the structural phase transition under high pressure is influenced by *s*-*d* hybridization from lower pressure to higher pressure phases^3^. At ambient pressure, As possesses a rhombohedral As-type (As-I) structure and transforms into a simple-cubic (As-II) structure at 25 GPa. At higher pressure, it transforms into a body-centered monoclinic host-guest (As-III) structure at 48 GPa, and then into a body-centered cubic (As-IV) at 97 GPa. Moreover, As is also presented by experimental study with superconducting transition temperature (*T*_*c*_) of 2.5 K and 32 GPa^4^.

Several metals are superconducting at high pressure^5–10^, with increasing pressure, it indicates that the superconducting transition temperature (*T*_*c*_) is also increasing. A striking example of the group-II elements, Ca has been reported highest *T*_*c*_ among elemental metals, with a *T*_*c*_ of 29 K at 216 GPa^5^. Sr and Ba have been reported to possess a *T*_*c*_ of 8 K at 58 GPa^6^, and a *T*_*c*_ of 5 K at 18 GPa.^7^, respectively. Moreover, the group-III elements, the *T*_*c*_ of Y has reached of 20 K at 115 GPa.^8,9^ and Sc has also observed to possess a *T*_*c*_ of 19.8 K at 107 GPa^9^. Furthermore, the group-V element, As has reached a maximum *T*_*c*_ of 2.7 K at 24 GPa^10^, Sb has reported to possess a *T*_*c*_ of 3.6 K at 3.5 GPa^10^, and Bi has reported to possess the highest *T*_*c*_ among the group-V element, with the *T*_*c*_ of 8.7 K at 9 GPa^10^. Recently, Chan *et al.*^11^ predicted the *T*_*c*_ of the simple cubic (sc) structure in the pressure range 30–50 GPa with the *T*_*c*_ as high as 2.99 K at 30 GPa. They also reported that the *T*_*c*_ of the sc structure decreases monotonically with increasing pressure *T*_*c*_ at 0.58 K and 50 GPa.

An important and a fundamental question remains to predict a new phase of As in the pressure range 100–300 GPa because there is no high-pressure phase beyond the bcc structure^2^. Thus, the searching technique leads to the discovery of high-pressure As structure. In addition, there is no reported the *T*_*c*_ for the predicted structure in As. It would be interesting to investigate the role of electron-phonon coupling (EPC) in order to predict *T*_*c*_ at high pressure.

In this work, in order to understand the predicted structure at high pressures, it is important to obtain as much information as possible about the structure. Hence, there have also been opened questions for As under extreme compression: (i) what is a new structure beyond the bcc structure at high pressure? (ii) Is the metallic high-pressure phase a superconductor? In order to address these problems, we report a theoretical investigation of the theoretical prediction which presents a new lowest enthalpy phase of As.

## Results and Discussion

**Figure 1:**
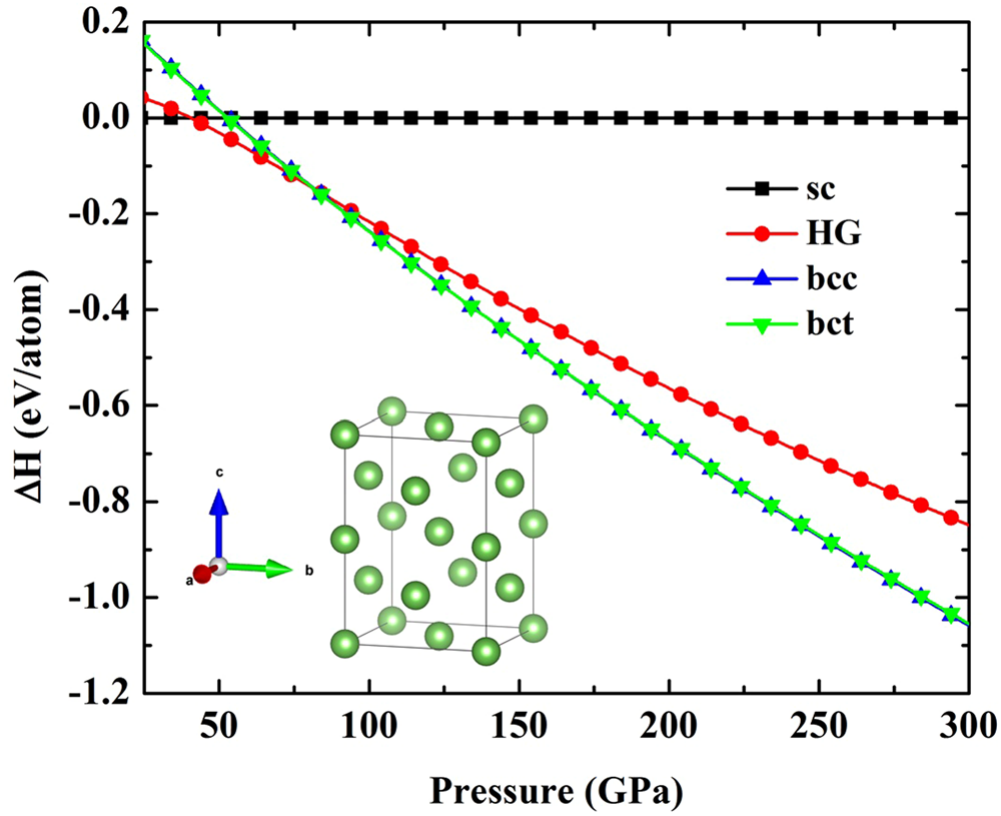
Comparison of the enthalpies of As phases up to 300 GPa. As structures relative to the *sc* structure at 0 K.

Our calculation has searched at pressures above 100 GPa, we find the lowest enthalpy structure of As which is the body-centered tetragonal (bct) structure with spacegroup I4_1_/acd. We also show that the simulated crystal structure of the bct structure is obtained as implemented in VESTA^12^ (Fig. 1). The optimized structural parameters for the bct structure are *a* = 4.072 Å and *c* = 5.802 Å with As atoms located at 8 *a* symmetry site (0, 0.5, 0.25). In Fig. 1, we present the enthalpies of different As phases with respect to the sc structure which are plotted as a function of pressure. Crossing points of curve from each structure represent the sc structure transforms into the HG structure at 41 GPa and into the bct structure at 81 GPa as shown in Fig. 1.

With increasing pressure, the transition sequence is successfully observed by the experimental study^1^. The low-pressure sc structure of As transforms into the monoclinic host-guest (HG) structure at pressure 48 GPa, and then into the body-centered cubic (bcc) structure at pressure 97 GPa^1^. On the contrary, our calculation proposes that transition sequence should be from the sc structure transforms into the HG structure at pressure 41 GPa and then into the bct structure at 81 GPa. Moreover, we show that the bcc and bct structures of As are very close in enthalpy which the bct structure has only slightly lower enthalpy than the bcc structure by 1 meV/atom.

Interesting, the bcc structure is the super-spacegroup (*Im*$\overline{{\rm{3}}}$*m*) and the bct structure is sub-spacegroup (*I*4_1_/*acd*) of the bcc structure which suggestes that the bct structure is a possible coexistence phase with the bcc structure. Our enthalpy calculation reveal that both the bcc and bct structures are remarkably closed overpressure range 100 to 300 GPa.

**Figure 2:**
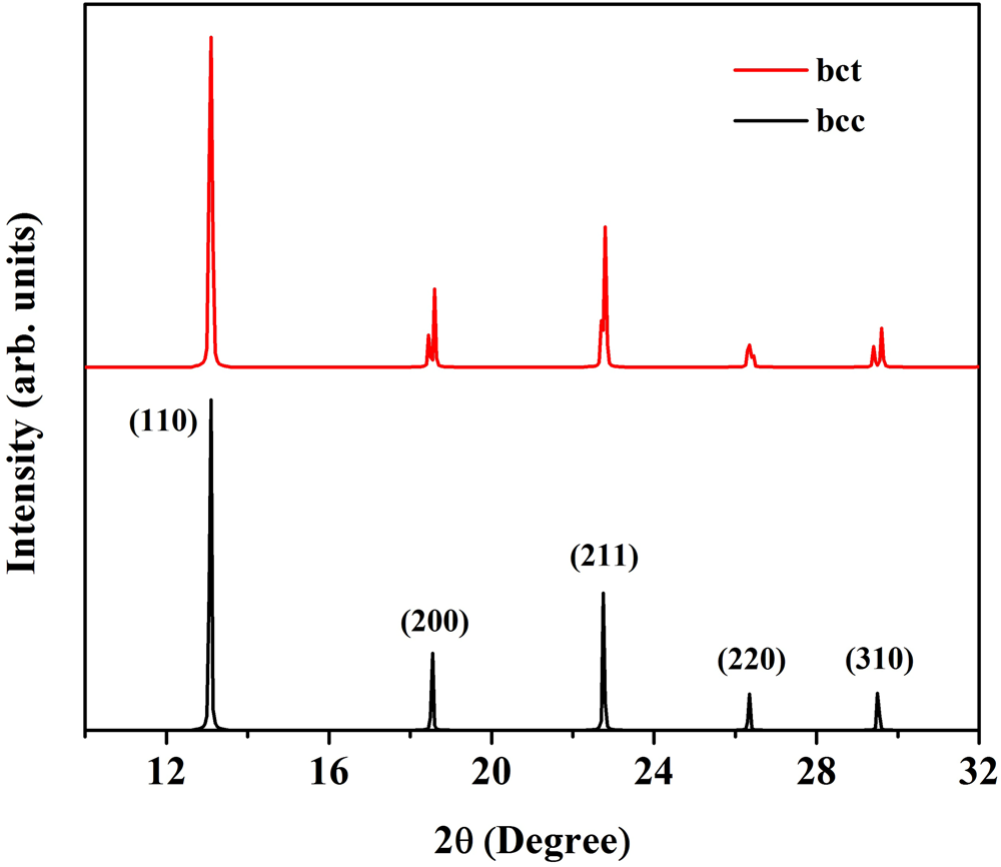
Simulated x-ray powder diffraction patterns of the bcc structure (black) and the bct structure (red).

In fact, the bcc and bct structures have very similar crystal structures. In order to determine difference between the bcc and bct structures, Fig. 2 shows a comparison of crystal structures by simulating powder diffraction patterns at 100 GPa. The peak 200, 211, 220, and 310 of the bct structure are distorted from the bcc structure because the bct index peaks split into two peaks with respect the bcc structure. The bct has been confirmed with slightly distorted parameter a/c lowered by 1% with respect to the bcc structure.

**Figure 3:**
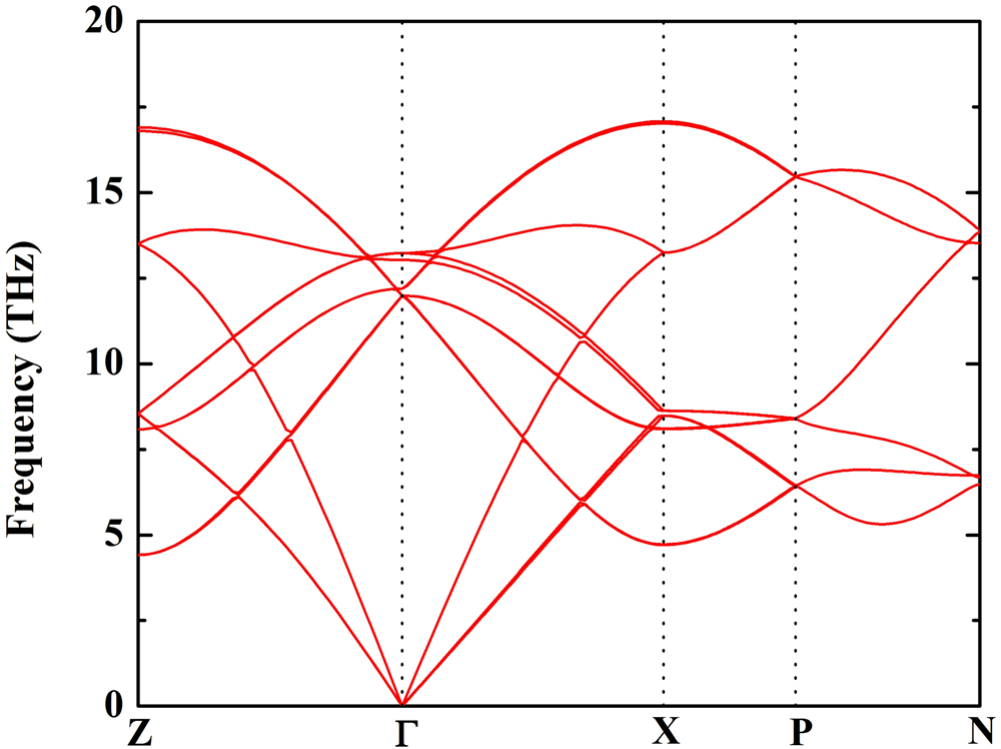
The dynamical harmonic stabilization of the bct structure at pressure 300 GPa.

This calculation leads to a new stable structure, we identify the dynamic stability of the bct structure by the phonon dispersion relation. The lack of imaginary frequencies provides a confirmation that the bct structure is stable at pressure 300 GPa (Fig. 3).

**Figure 4:**
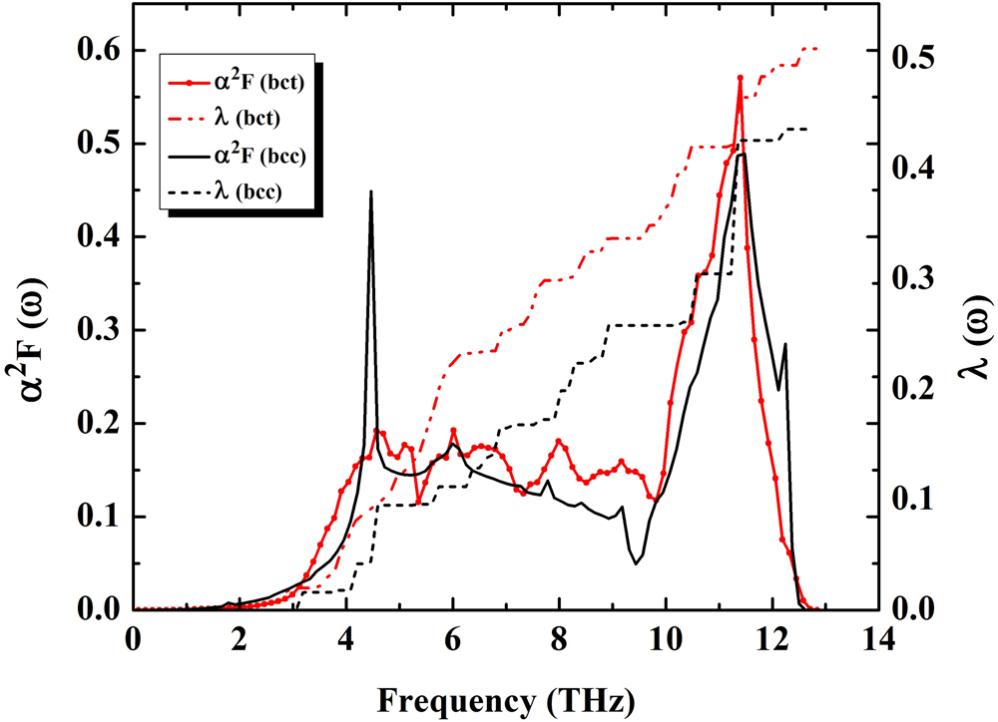
Spectral function *α*^2^*F*(*ω*) and integrated *λ* as a function of frequency of As at pressure 150 GPa.

**Figure 5:**
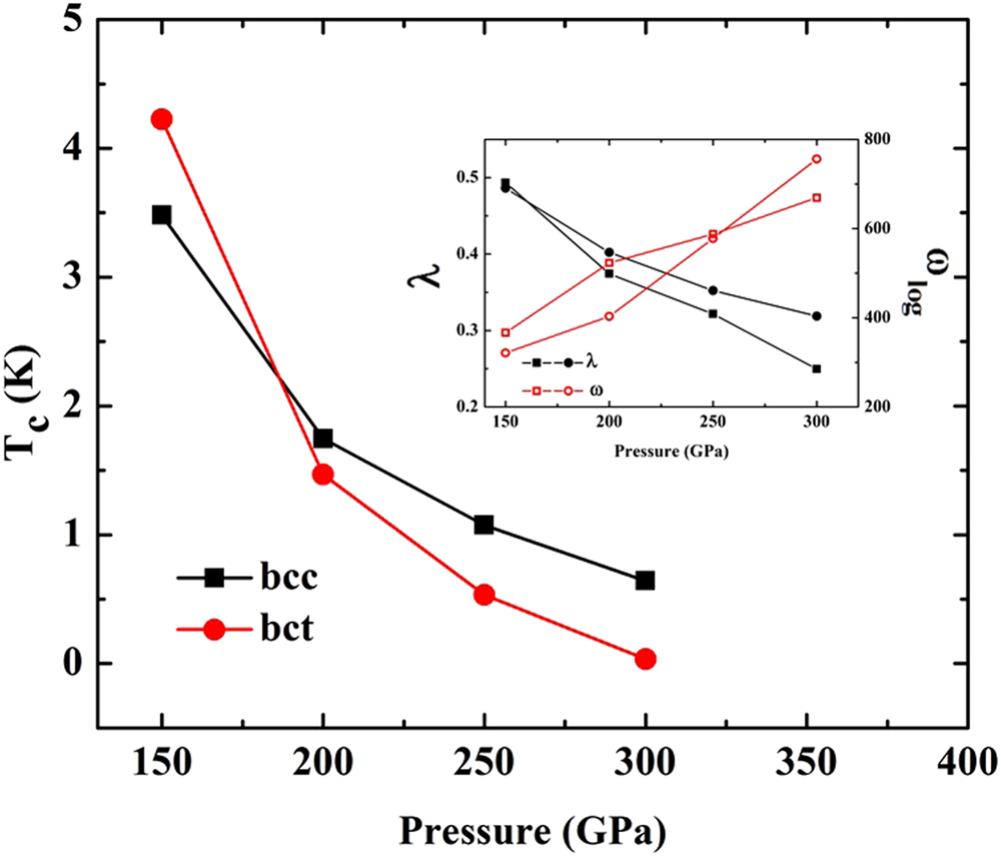
Superconducting *T*_*c*_ of As as a function of pressure. The square and the circle symbols represent calculations for the bcc and bct structures, respectively. The inset shows calculated electron-phonon coupling for the bcc structure (filled circle) and the bct structure (filled square) and averaged phonon frequency as a function of pressure for the bcc structure (hollow circle) and the bct structure (hollow square).

In the whole pressure interval of 150–300 GPa, we use a fixed value for the effective Coulomb interaction parameter. It is worth noting that the value of effective Coulomb interaction parameter also used μ^*^ = 0.10, which is assumed for all metals by Allen and Dynes^13^. In fact, the μ^*^ plays an important role to give the *T*_*c*_ at high pressure. Especially, the nature of As is observed that the *T*_*c*_ is decreased with increasing pressure through the evolution of μ^*^^11^. We also explored the effects on *T*_*c*_ of changing μ^*^ from 0.10 to 0.18^13–15^ at 150 GPa as shown in Table 1 Thus, we did consider the effect of μ^*^ = 0.10 on the predicted *T*_*c*_ because it gives the highest *T*_*c*_ among the evolution of μ^*^. Our prediction concerning the superconducting phase of As at high pressures would be experimentally confirmed. At 150 GPa, the spectral function *α*^2^*F* of the bct structure is higher than those of the bcc structure around frequency region 6–13 THz. Likewise, the integrated *λ* of the bct structure is also higher than the bcc structure (Fig. 4). Moreover, it exhibits the metallization and it predicts to give temperature superconductivity in the bct structure, with the *T*_*c*_ of 4.2 K at 150 GPa as can be seen Fig. 5.

**Table 1:** Calculated effective Coulomb interaction parameter μ^*^, and superconducting transition temperature *T*_*c*_ for As at 150 GPa.

Structures	Tc (K)			
	μ^*^ = 0.10	μ^*^ = 0.13	μ^*^ = 0.15	μ^*^ = 0.18
bct	4.2	2.5	1.6	0.7
bcc	3.4	2.0	1.3	0.5

At high pressure, pressure-induced superconductivity shown in Fig. 5. We also explore the effects on *T*_*c*_ of increasing pressure. Our calculations reveal *T*_*c*_ both the bcc and bct structures with increasing pressure, which generally shows monotonic decreasing behaviours. Especially, the *λ* of the bct structure is higher than the bcc structure at 150 GPa which has led to suggestions for possible the highest *T*_*c*_ of the bct structure. It indicates that the *T*_*c*_ of the bct structure is higher than the bcc structure, where it possesses the highest *T*_*c*_ of 4.2 K.

Above 150 GPa, we find that the *T*_*c*_ of the bct structure is lower than the bcc structure as well as *T*_*c*_ gradually decreases with pressure. We calculate *λ* and the averaged phonon frequency (*ω*_*log*_) as a function of pressure of the bcc and bct structures. The *λ* of the bcc and bct structures decrease monotanically between150 GPa and 300 GPa, respectively. The *ω*_*log*_ of the bcc and bct structures increase monotonically between 150 GPa and 300 GPa, respectively as can be seen the inset in Fig. 5.

Moreover, we strongly suggested that the bct structure is not superconducting phase above 300 GPa. This is due to the fact that the bct structure remains in the metallic state at 300 GPa, we propose that it becomes a normal metallic state since the *λ* of the bct structure is poorly characterized, with increasing pressure. It should be noted that the *λ* is weakly coupling for calculation the *T*_*c*_. Here again, the μ^*^ plays a key role, in the sense that the increasing pressure induces the *T*_*c*_ of the bct structure decreasing. This might lead to new interpretation of the experimental data above 150 GPa.

## Conclusion

In conclusion, the AIRSS technique reveals the new stable bct structure with spacegroup I4_1_/acd of As at high pressure. We show that structural phase transformation of As from sc to HG to bct at 41, and 81 GPa, respectively. We confirme that bct is themodynamically stable above 81 GPa and our result show that it is also dynamically stable at 300 GPa. Therefore, we suggest the transitions sequence from the sc structure transforms into the HG structure at 41 GPa and then into the bct structure at 81 GPa. We find that the *T*_*c*_ of the bct structure is the maximum around 4.2 K at 150 GPa. It is worth noting that from our calculation both bct and bcc structures share remarkable similarity in structural and property. Thus, both structures may be observed by experimental at low temperature and high pressure.

## Methods

We used *ab initio* random structure searching (AIRSS) technique^16,17^ and *ab initio* calculation of the Cambridge Serial Total Energy Package (CASTEP)^18^ to predict the candidate crystal structures of As under pressure. The AIRSS technique is used to predict the crystal structures of materials^16,17,19–22^. A plane-wave basis-set energy cutoff of 280 eV and an initial Brillouin-zone (BZ) sampling grid of spacing 2 *π*  × 0.07 Å^−1^ were used for this calculation. The generalized gradient approximation (GGA) with the Perdew-Burke-Ernzerhof (PBE) parametrization^23^ for the exchange-correlation functional was used for the structure searching. The AIRSS technique generated unit cells of random shapes with reasonable volumes.

We studied simulation cells containing 2, 4, 6, 8, 10, and 12 atoms of As at pressure 100, 150, 200, 250, and 300 GPa. The cell shapes and atomic positions are then relaxed to the ground state structure at each pressures. The AIRSS technique calculated the enthalpies of the phases at any pressure by using the simple linear approximation^17^.

1 $$H(p)\simeq H({p}_{s})+{V}_{s}(p-{p}_{s}),$$

Where *p* is any pressure and *V*_*s*_ is the volume of the phase at *p*_*s*_. The quantities *H*(*p*_*s*_), *p*_*s*_ and *V*_*s*_ are calculated for each relaxed into higher-symmetry space groups obtained in a search.

We are here presented electronic structure by using the GGA-PBE^23^ for the exchange-correlation functional to density functional theory. We employed the projector augmented wave (PAW) method^24^, as implemented in the Vienna ab initio simulation package (VASP)^25^. The PAW potential with a 15-electron (3 *d*^10^4 *s*^2^4 *p*^3^) for As has been employed with plane waves basis set up to a cutoff energy of 500 eV and the initial BZ sampling grid of spacing 2 *π*  × 0.02 Å.

All structures were relaxed and their equations of state were obtained by fitting to the calculated energy-volume data with the third-order Birch-Murnaghan equation. We then calculated the enthalpy-pressure relationship and the high pressure phase from the experimental information^1^.

The phonon dispersion of candidate structure was calculated by *ab initio* lattice dynamics with finite displacement method, as implemented in VASP code and the phonopy package^26^.

We calculated the EPC with density functional perturbation theory^27^. The plan waves basis set was expanded with a kinetic energy cutoff of 60 Ry. The calculation studies presented here are based on the GGA-PBE. We employed PAW method as implemented in Quantum Espresso^28^. The BZ integrations in the electronic and phonon calculations were performed using MP meshes. Both the meshes of k-points for electronic states and the meshes of phonons were used in these calculation. For As-IV, individual phonon calculations were performed on the first BZ on 4 × 4 × 4 q-meshes with a 12 × 12 × 12 k-points mesh. For high-pressure candidate structure, individual phonon calculations were performed on the first BZ on 4 × 4 × 4 q-meshes with a 8 × 8 × 8 k-points mesh. For As-IV, The EPC matrix elements were computed in the first BZ on 4 × 4 × 4 q-meshes using individual EPC matrices obtained with a 24 × 24 × 24 k-points mesh. For the bct structure, The EPC matrix elements were computed in the first BZ on 4 × 4 × 4 q-meshes using individual EPC matrices obtained with a 16 × 16 × 16 k-points mesh. We calculated *T*_*c*_ using the Allen-Dynes equation^13^, which is corresponded for *λ* < 1.4,

2 $${T}_{c}=\frac{{\omega }_{log}}{1.2}\exp [-\frac{\mathrm{1.04(1}+\lambda )}{\lambda -{\mu }^{\ast }\mathrm{(1}+0.62\lambda )}],$$

where *ω*_*log*_ is the averaged phonon frequency. We used effective Coulomb interaction parameter μ^*^ = 0.10. It is assumed by the original Allen-Dynes formula^13^.
